# Hierarchically Structured Nb_2_O_5_ Microflowers with Enhanced Capacity and Fast-Charging Capability for Flexible Planar Sodium Ion Micro-Supercapacitors

**DOI:** 10.1007/s40820-023-01281-5

**Published:** 2024-01-04

**Authors:** Jiaxin Ma, Jieqiong Qin, Shuanghao Zheng, Yinghua Fu, Liping Chi, Yaguang Li, Cong Dong, Bin Li, Feifei Xing, Haodong Shi, Zhong-Shuai Wu

**Affiliations:** 1 State Key Laboratory of Catalysis, Dalian Institute of Chemical Physics, Chinese Academy of Sciences, 457 Zhongshan Road, Dalian, 116023 People’s Republic of China; 2 School of Materials Science and Engineering, Zhengzhou University, Zhengzhou, 450001 People’s Republic of China; 3 College of Science, Henan Agricultural University, No. 63 Agricultural Road, Zhengzhou, 450002 People’s Republic of China; 4 Dalian National Laboratory for Clean Energy, Chinese Academy of Sciences, 457 Zhongshan Road, Dalian, 116023 People’s Republic of China; 5 University of Chinese Academy of Sciences, 19A Yuquan Road, Shijingshan District, Beijing, 100049 People’s Republic of China; 6 Hebei Key Lab of Optic-Electronic Information and Materials, The College of Physics Science and Technology, Institute of Life Science and Green Development, Hebei University, Baoding, 071002 People’s Republic of China

**Keywords:** Nb_2_O_5_ nanosheets, Microflowers, Sodium ion micro-supercapacitors, Flexibility, Energy storage

## Abstract

**Highlights:**

- Hierarchically structured Nb_2_O_5_ microflowers consiste of porous and ultrathin nanosheets.
- Nb_2_O_5_ microflowers exhibit enhanced capacity and rate performance boosting Na ion storage.
- Planar NIMSCs with charge and kinetics matching show superior areal capacitance and lifespan.

**Abstract:**

Planar Na ion micro-supercapacitors (NIMSCs) that offer both high energy density and power density are deemed to a promising class of miniaturized power sources for wearable and portable microelectronics. Nevertheless, the development of NIMSCs are hugely impeded by the low capacity and sluggish Na ion kinetics in the negative electrode. Herein, we demonstrate a novel carbon-coated Nb_2_O_5_ microflower with a hierarchical structure composed of vertically intercrossed and porous nanosheets, boosting Na ion storage performance. The unique structural merits, including uniform carbon coating, ultrathin nanosheets and abundant pores, endow the Nb_2_O_5_ microflower with highly reversible Na ion storage capacity of 245 mAh g^−1^ at 0.25 C and excellent rate capability. Benefiting from high capacity and fast charging of Nb_2_O_5_ microflower, the planar NIMSCs consisted of Nb_2_O_5_ negative electrode and activated carbon positive electrode deliver high areal energy density of 60.7 μWh cm^−2^, considerable voltage window of 3.5 V and extraordinary cyclability. Therefore, this work exploits a structural design strategy towards electrode materials for application in NIMSCs, holding great promise for flexible microelectronics.
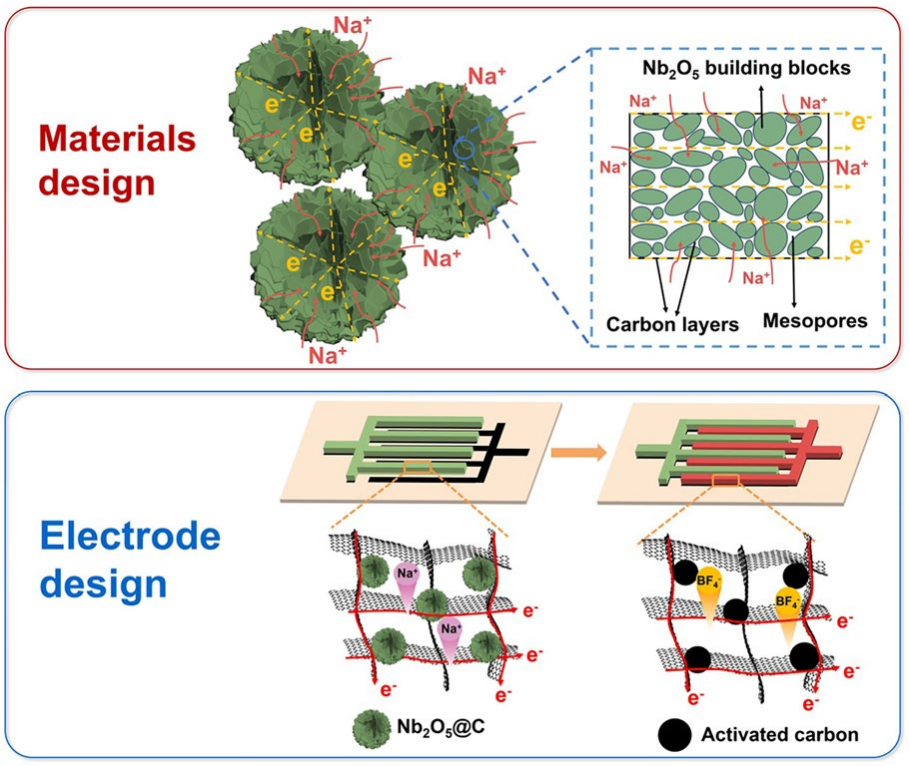

**Supplementary Information:**

The online version contains supplementary material available at 10.1007/s40820-023-01281-5.

## Introduction

With the booming development of emerging wearable and portable electronics, such as foldable smartphones, shape-conformable healthy monitors and wearable sensors, the flexible and miniaturized energy storage systems are expected to offer excellent energy and power density as well as long lifespan [1–5]. A hybrid ion micro-supercapacitor is made up of a battery-type anode and a capacitor-type cathode, which combines the benefits of battery and supercapacitor and achieves a balance between energy density and power density [6–13]. In this regard, sodium-ion micro-supercapacitors (NIMSCs) are deemed to a highly competitive class of next-generation miniaturized energy storage devices due to more earth-abundant sodium source and its low cost [14–17]. According to the working mechanism of sodium ion capacitors, the battery-type anodes have been reported for enhancing Na ion storage performance, including high capacity (*e.g.*, RuO_2_, Ti_3_C_2_T_*x*_, Ti_2_C ) [18–20] and decent rate performance (e.g., TiO_2_, NaTi_2_(PO_4_)_3_, Na_3_V_2_(PO_4_)_3_) [21–24] and novel polymer materials [25–27], towards well capacity and/or kinetics matching with capacitor-type cathodes. Unfortunately, the attention is rarely focused on simultaneously fortifying the capacity and rate capability of anode materials. Notably, pseudocapacitive materials possess rapid redox reaction and excellent Na ion storage performance, providing a feasible way to address the trade-off between capacity and kinetics [28, 29].

Recently, the orthorhombic niobium pentoxide (*T*-Nb_2_O_5_) with high chemical stability and open framework, exhibiting intercalation pseudocapacitance behavior, has been regarded as a key high-rate anode material for Na ion storage [30–32]. Moreover, its large lattice spacing (3.9 Å) corresponding to (001) plane is conducive to fast Na ion (2.04 Å) transport [33, 34]. However, the intrinsic poor electronic conductivity of Nb_2_O_5_ makes it sluggish reaction kinetics, further resulting in low rate capability and cycling performance. So far, several strategies have been applied to boost reaction kinetics, *e.g.*, preparing conductive Nb_2_O_5_ composites with carbon species [35, 36], and forming porous networks to shorten ion transfer pathway [37, 38]. Despite all the designs through combining with high conductive carbon, they are generally the aggregated powders or irregular nanoparticles, far away from the excellent rate capability and ultralong cycling stability for NIMSCs [39, 40]. It should be mentioned that three-dimensional (3D) hierarchical micro/nanostructures based on elaborate building blocks not only present favorable structural stability, but also show shortened electron/ion diffusion pathway [41, 42]. However, the facile fabrication of Nb_2_O_5_ with desirable hierarchical architectures and high electrochemical activity remains significant challenges.

In this work, we reported the hierarchical carbon-coated Nb_2_O_5_ microflowers consisted of ultrathin porous nanosheets, serving as negative electrode coupled with actived carbon (AC) positive electrode for planar high-performance NIMSCs with interdigital microelectrodes operated in a high-voltage ionogel electrolyte. The uniform carbon layer onto Nb_2_O_5_ enhanced the electron migration and structural stability of microelectrodes, and the hierarchical microflower structure enabled the sufficient electrolyte contact, shortening ion/electron diffusion pathways. As a consequence, the Nb_2_O_5_ microflowers delivered a highly reversible capacity of 245 mAh g^−1^ at 0.25 C and good rate capability (122 mAh g^−1^ at 20 C) for Na ion storage. Because of well matched kinetics between high-rate Nb_2_O_5_ microflowers and capacitor-type AC, the as-fabricated NIMSCs showed high areal capacitance of 41 mF cm^−2^ at 50 μA cm^−2^, exceptional long-term cyclability and high areal energy density of 60.7 μWh cm^−2^, exceeding the mostly reported microsupercapacitors [43–45].

## Experimental Section

### Synthesis of Nb_2_O_5_ Mciroflowers

The Nb_2_O_5_ mciroflowers were prepared via a controllable hydrothermal method and subsequent annealing treatment. First, niobium oxalate hydrate (1 mmol), was dissolved in a mixed solution of ethylene glycol (30 mL) and water (50 mL). Second, the mixture was further stirred for 1 h to obtain homogenous and transparent solution, in which 0.4 mL of ammonium hydroxide was used to adjust the pH to neutral value. Afterwards, the obtained solution was hydrothermal treated at 180 °C for 24 h in a 100 mL steel autoclave. Subsequently, the resultant precursors under a heating rate of 5 °C min^−1^ were annealed at 650 °C (or 500 and 800 °C) for 5 h in air, resulting in Nb_2_O_5_ microflowers (NF-500, NF-650 and NF-800). For the preparation of the carbon-coated Nb_2_O_5_ microflowers, the obtianed NF-650 (60 mg) was added into dopamine (30 mg) and tris-base (40 mg) solution (50 mL) under stirring for 6 h. Finally, the carbon-coated Nb_2_O_5_ microflowers (NF@C-650) were achieved by annealing polydopamine-coated precursors at 600 °C (5 °C min^−1^) for 3 h in Ar atmosphere.

### Ionogel Electrolyte

NaBF_4_ was first dissolved into 1-ethyl-3-methylimidazolium tetrafluoroborate (EMIMBF_4_) to form 1.0 M NaBF_4_-EMIMBF_4_ solution. Then, poly(vinylidenedifluoride-cohexafluoropropylene (PVDF-HFP) acetone dispersion (3 mL) was mixed with the obtained solution under continuous stirring for 2 h. After that, NaBF_4_-based ionogel electrolyte of NaBF_4_-EMIMBF_4_-PVDF-HFP was obtained.

### Fabrication of NIMSCs

The NIMSCs were obtained through a mask-assisted filtration method [7, 46], in which a stainless-steel mask with interdigitated pattern with eight fingers (four fingers at each side) was used. First, 1 mL electrochemcially exfoliated graphene (EG) dispersion (0.1 mg mL^−1^) in ethanol was filtrated at the both sides of the mask on a nylon membrane. Then, Nb_2_O_5_ and AC dispersion (0.5 mg mL^*−*1^) was injected into the each side of the same mask, forming negative electrode and positive electrode, respectively. Afterwards, 2 mL dilute EG dispersion (0.02 mg mL^*−*1^) was continuously filtrated onto the electrodes. Fianally, after a pressure of 20 MPa via the pressure machine carried out on filtrated electrodes, the NIMSCs were achieved.

## Results and Discussion

### Fabrication of Nb_2_O_5_ Microflowers for NIMSCs

**Fig. 1:**
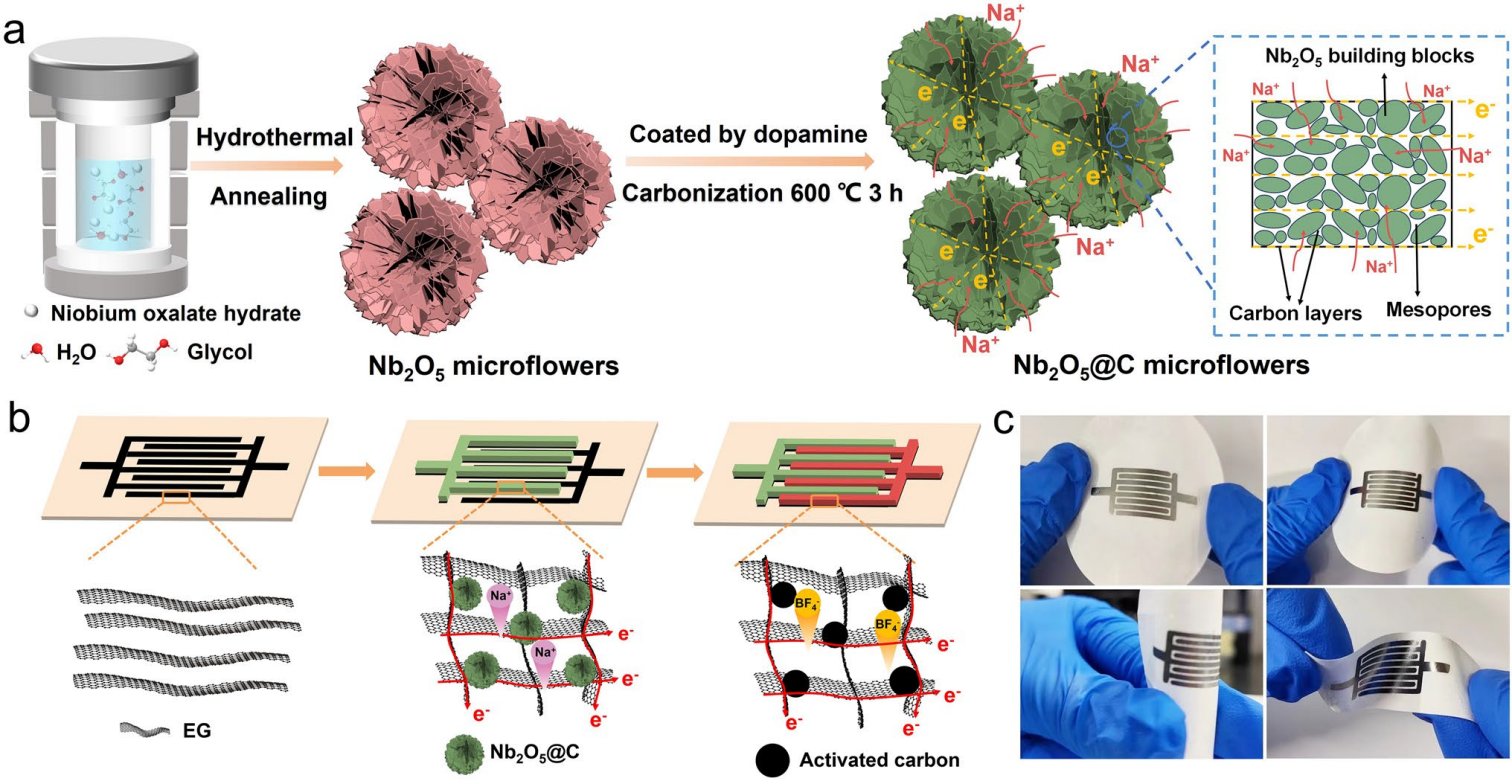
Schematic illustration of the synthesis process of Nb_2_O_5_ microflowers and planar NIMSCs. **a** Preparation process of carbon-coated Nb_2_O_5_ microflowers via hydrothermal method. Nb_2_O_5_ microflowers with fast electron/Na ion charge channels and high contact area. **b** Fabrication process of planar NIMSCs through mask-assisted filtration method, exhibiting highly conductive network for ion and electron transport. **c** Photographs of the as-fabricated NIMSCs

The synthesis process of the Nb_2_O_5_ microflowers was schematically illustrated in Fig. 1a. Firstly, hierarchical Nb_2_O_5_ microflowers were achieved by combining hydrothermal treatment with subsequent annealing process in air. Then, polydopamine serving as the carbon and nitrogen sources were uniformly coated onto the as-obtained Nb_2_O_5_ microflowers. Finally, the resulting carbon-coated and nitrogen-doped Nb_2_O_5_ microflowers (NF@C-650) with ultrathin nanosheets and abundant in-plane pores were achieved by annealing at 600 °C in inert atmosphere. The as-prepared Nb_2_O_5_ microflowers were used as negative electrode coupled with AC as positive electrode to fabricate planar flexible NIMSCs (Fig. 1b). Highly conductive EG nanosheets served as the current collectors and conductive additive. The current collector, anode and cathode were successively deposited on nylon membrane via mask-assisted filtration method [46], in which EG nanosheets played a key role on two-dimensional conductive pathway in microelectrodes, forming long-range-ordered electron channels. Applying bent and twisted stress on NIMSCs, they could hold well structural integrity and mechanical flexibility without any delamination for microelectrodes (Fig. 1c).

### Characterization of Nb_2_O_5_ Microflowers

**Fig. 2:**
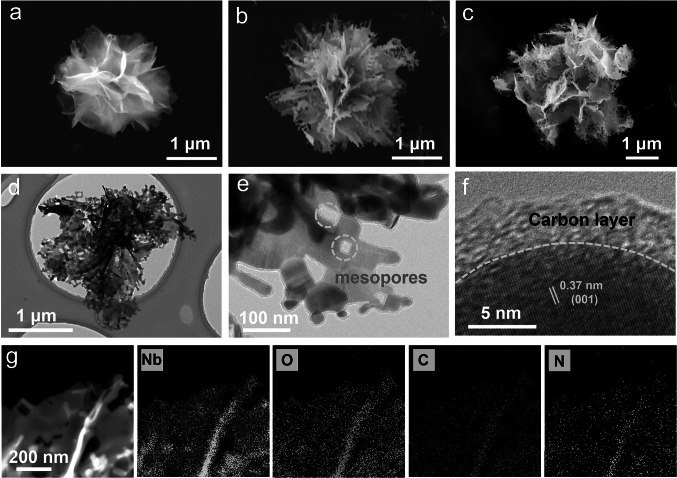
Morphology characterization of Nb_2_O_5_ microflowers. **a-c** SEM images of Nb_2_O_5_ precursor (**a**), NF-650 (**b**) and NF@C-650 (**c**). **d, e** TEM images of NF@C-650 with different magnifications. **f** HRTEM image of NF@C-650. **g** STEM image and corresponding EDS mappings of NF@C-650

The evolution process of porous Nb_2_O_5_ microflower was carefully elucidated by temperature-dependent annealing conditions. The resultant samples via different annealing temperature (*i.e.*, 500, 650, and 800 °C) were denoted as NF-500, NF-650 and NF-800, respectively. The Nb_2_O_5_ precursors showed a regular microflower-like structure with an average diameter of ~ 3 µm (Fig. 2a). . With the annealing treatment, NF-500 maintained the primary intact microflower morphology (Fig. S1), but a small number of impurity phase of precursor was still observed in XRD pattern and Raman spectra (Fig. S2). As the annealing temperature increased to 650 °C, the porous Nb_2_O_5_ microflowers (NF-650) were obtained (Fig. 2b, c). Polydopamine serving as the carbon and nitrogen sources were uniformly coated onto the as-obtained  NF-650 (Figs. S3-S4), in which carbon-coated  Nb_2_O_5_ microflowers (NF@C-650) were achieved by annealing at 600 °C in inert atmosphere. Both NF-650 and NF@C-650 could well retain the morphology of the precursors with hierarchical microflowers composed of the vertically intercrossed and porous nanosheets with ultrathin thickness of ~ 30 nm. The XRD patterns of NF-650 and NF@C-650 apparently exhibited the sharp and intense diffraction peaks, confirming orthorhombic-phase (JCPDS NO. 30–0873) [47, 48] and highly crystalline Nb_2_O_5_ without impurities (Fig. S2a). As shown in Raman spectra (Fig. S2b), the broad peak (~ 690 cm^−1^) and two characteristic peaks (228 and 302 cm^−1^) are ascribed to Nb_2_O_5_ [49], and two obviously peaks located at ∼1360 cm^−1^ (*D*-band) and ∼1595 cm^−1^ (*G*-band) certified the carbon species on the surface of NF@C-650 [50], in which the carbon content was about 7.8 wt% according to thermogravimetry analysis (Fig. S5). In addition, both the Nb 3*d*, O 1* s* XPS spectra and EPR curve identified the existence of abundant oxygen vacancies in NF@C-650 (Fig. S6). The N_2_ adsorption and desorption isotherm displayed characteristic type IV curve with a hysteresis loop, indicative of the mesoporous feature (Fig. S7). The corresponding pore size distribution curve presented average pore sizes at 3.9 nm, which was consistent with TEM observation (Fig. 2d, e). The mesopores may derive from the volatilization of organic species at high temperature. Clearly, a thin carbon layer of ~ 5 nm was uniformly coated onto the surface of Nb_2_O_5_ (Fig. 2f). As shown in high-resolution TEM (HRTEM) image (Figs. 2f and S8), NF@C-650 displayed the lattice fringe of 0.37 nm corresponding to the (001) crystal face of *T*-Nb_2_O_5_. Due to the coating of polydopamine, nitrogen heteroatom was brought into the NF@C-650 apart from carbon species. The element mappings of NF@C-650 showed homogeneous distribution of Nb, O, C, N elements in comparison with NF-650 (only Nb and O elements) (Figs. 2g and S9). Once the annealing temperature was over 800 °C, the flower-like Nb_2_O_5_ was fused (NF-800), in which the ultrathin nanosheets were agglomerated into irregular columnar shape, exhibiting actiniae-like morphology (Fig. S10). Thus, the optimal annealing temperature for high-crystallized *T-*Nb_2_O_5_ microflower was 650 °C.

### Electrochemical Performance of Nb_2_O_5_ Microflowers

**Fig. 3:**
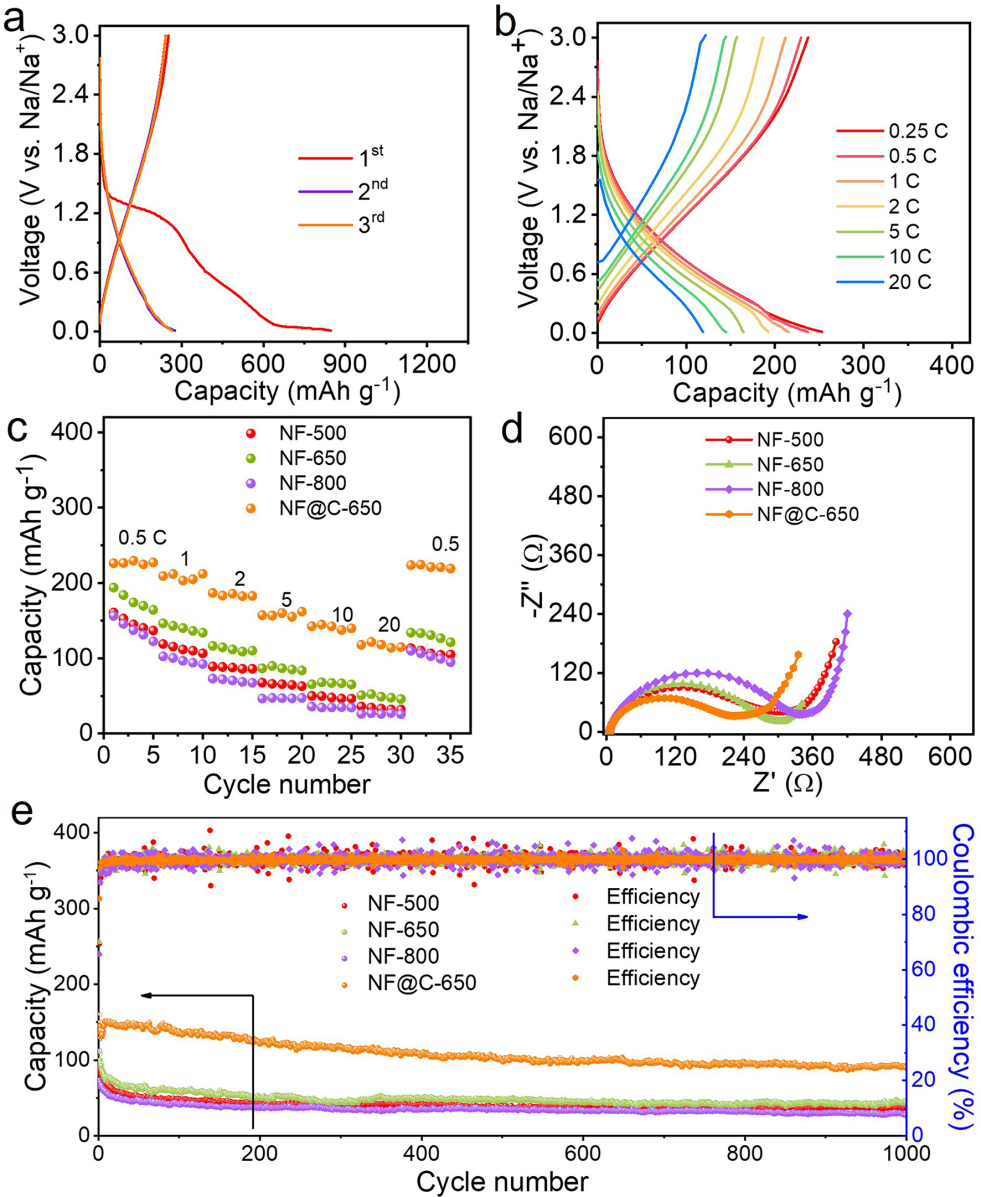
Electrochemical performance of Nb_2_O_5_ microflowers for Na ion storage at a potential range of 0.01–3.0 V (*vs.* Na/Na^+^). **a, b** GCD profiles of NF@C-650 tested at 0.25 C (**a**) and different rates of 0.25–20 C (**b**). **c-e** Rate capability (**c**), EIS plots (**d**) and cycling performance at 20 C (**e**) of various Nb_2_O_5_ microflowers

The sodiation and de-sodiation behaviors of Nb_2_O_5_ microflowers were evaluated in half cells in NaClO_4_ electrolyte under the potential range of 0.01–3.0 V (*vs*. Na/Na^+^) (Fig. 3). As shown in cyclic voltammetry (CV) curves (Fig. S11), two irreversible cathodic peaks located at ~ 1.06 and 0.3 V were observed in the first cycle and disappeared in the subsequent cycles, attributing to the side reactions and electrolyte decomposition, forming solid electrolyte interphase layers on the surface of NF@C-650. The NF@C-650 showed continuous slope-type profiles in the galvanostatic charge–discharge (GCD) profiles (Fig. 3a) corresponding to the broad cathodic and anodic peaks in the CV curves, indicative of dominant surface pseudocapacitive contribution [33]. Remarkably, NF@C-650 delivered a highly reversible capacity of 245 mAh g^−1^ at 0.25 C (1 C = 200 mA g^−1^), which was higher than those of other Nb_2_O_5_ microflowers tested at 0.25 C, such as NF-500 (160 mAh g^−1^), NF-650 (193 mAh g^−1^) and NF-800 (156 mAh g^−1^) (Fig. S12a), and extremely exceeding previous reported Nb_2_O_5_ materials for Na ion storage, such as Nb_2_O_5_ nanosheets (170 mAh/g at 50 mA g^−1^) [51], mesoporous Nb_2_O_5_ nanosheets (230 mAh g^−1^ at 0.25 C) [52]. More importantly, NF@C-650 showed excellent rate capability at varying current densities from 0.5 to 20 C (Fig. 3b, c), retaining a high capacity of 122 mAh g^−1^ at 20 C, witnessing its fast charging ability, far outperforming NF-500 (36 mAh g^−1^), NF-650 (52 mAh g^−1^) and NF-800 (27 mAh g^−1^) (Fig. S12b). To clarify the high capacity and rate capability of NF@C-650, electrochemical impedance spectroscopy (EIS) was analyzed (Fig. 3d). NF@C-650 exhibited a lower charge transfer resistance (*R*_*ct*_) of 217 Ω than NF-500 (322 Ω), NF-650 (302 Ω) and NF-800 (340 Ω). Moreover, NF@C-650 presented a steep slope at the low-frequency Warburg impedance region, revealing the fast ion diffusion behavior in compassion with other controlled samples. It is manifested that NF@C-650 with porous ultrathin nanosheets as building blocks could greatly boost the ion/electron transfer kinetics, thus possessing superior electrochemical performance. In addition, NF@C-650 showed exceptional long-term cycling performance, delivering a stable capacity of ~ 90 mAh g^−1^ accompanying by high Coulombic efficiency of almost 100% and maintaining well structure and flower-like morphology of NF@C-650 after 1000 cycles at 20 C (Figs. 3e and S13–S14), demonstrating excellent stability of hierarchically carbon-coated Nb_2_O_5_ microflower. Based on the above, it is worth noting that flower-like NF@C-650 with a uniform thin carbon layer exhibited well electrochemical performance for Na ion storage, primarily because of its combined advantages, including (i) ultrathin nanosheets with short ion/electron diffusion pathway, (ii) abundant pore structures providing high active surface area for fast electrolyte infiltration and Na ion transport, and (iii) highly conductive carbon layer boosting electron transfer kinetics and improving interface stability.

### Electrochemical Performance of NIMSCs

**Fig. 4:**
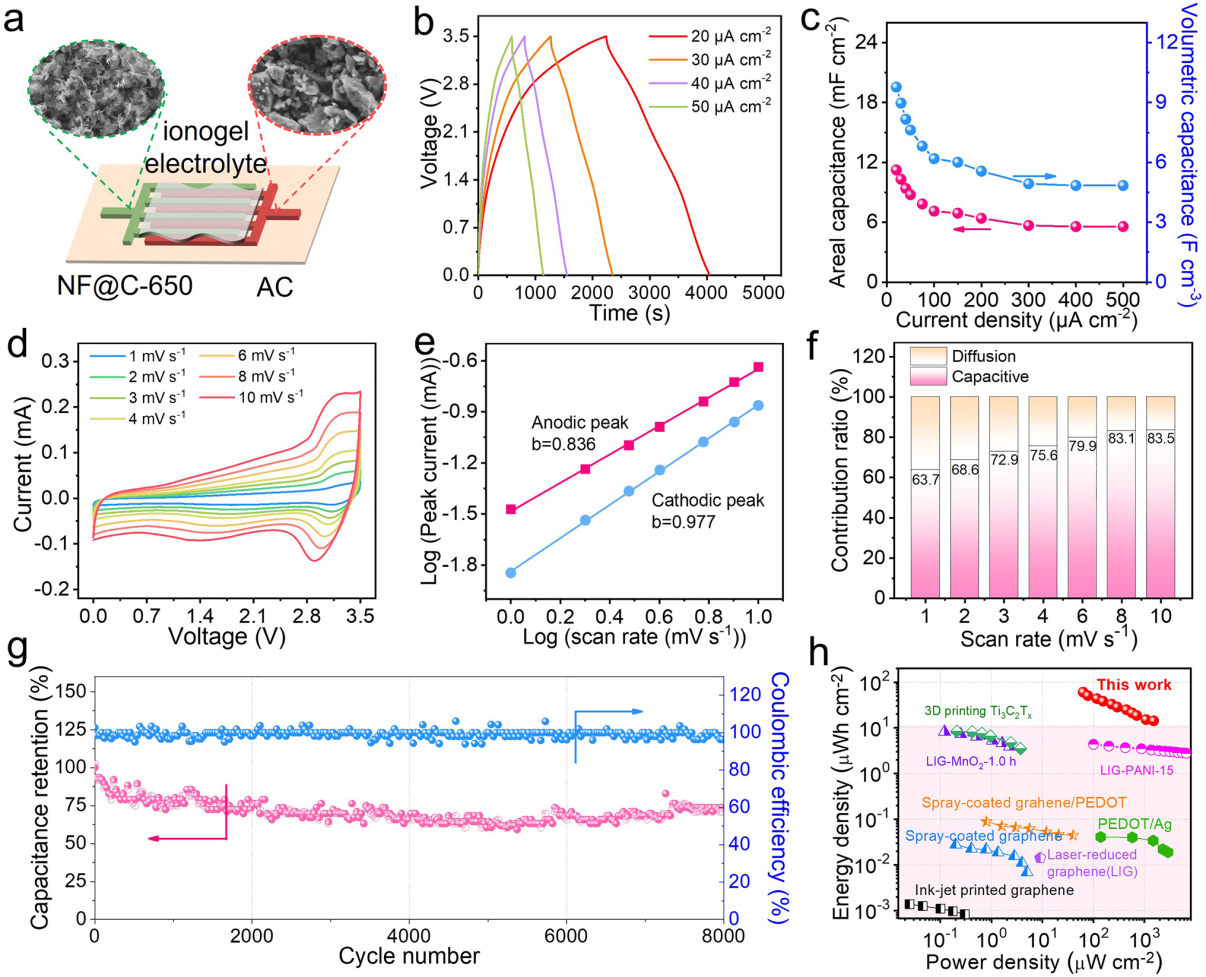
Electrochemical performance of NF@C-650//AC-NIMSCs. **a** Schematic diagram of ionogel-based NIMSCs and SEM images of two microelectrodes. **b** GCD profiles of NIMSCs measured at 20–50 μA cm^−2^. **c** Areal capacitance and volumetric capacitance of NIMSCs calculated from GCD profiles at 20–500 μA cm^−2^. **d** CV curves obtained at different scan rates from 1 to 10 mV s^−1^. **e** The plots between peak current and scan rate.** f** Normalized capacitance contribution consisted of capacitive and diffusion-controlled contribution at 1–10 mV s^−1^. **g** Long-term cycling performance of NIMSCs at 300 μA cm^−2^. **h** Ragone plot of NIMSCs compared with the reported micro-supercapacitors

To demonstrated the applicability of high-performance NF@C-650, we further assembled flexible planar NIMSCs using it as negative electrode and AC as positive electrode, where the EG nanosheets served as the conductive additive and current collector. As shown in Fig. 4a, the NF@C-650//AC-NIMSCs were constructed by patterning the asymmetric interdigital microelectrodes using mask-assisted filtration method and casting ionogel electrolyte of NaBF_4_-EMIMBF_4_-PVDF-HFP [16]. The resultant Nb_2_O_5_ negative electrode and AC positive electrode displayed a thickness of 10 and 13 μm (Fig. S15), respectively. It was clearly seen that the active electrode materials were compactly sandwiched in the highly conductive EG nanosheets used in the top layer (Fig. S16), bottom layer and interlaced intermediate layer, forming a 3D conductive electron transfer channel. As a result, the microelectrodes showed decent conductivity of ~ 42 S cm^−1^ (Fig. S17). Such the elaborate highly conductive NF@C-650 materials with high rate capability were well matched kinetics with typically high-rate capacitor-type AC, which was crucial for designing high-performance NIMSCs.

The electrochemical performance of as-fabricated NIMSCs were examined in high-ionic-conductivity NaBF_4_ based ionogel electrolyte (8.1 mS cm^−1^) at a voltage of 3.5 V [53]. As shown in CV curves (Fig. S18), it was observed that a pair of redox peaks between 2.8 and 3.4 V corresponding to the slope line in the GCD profiles (Fig. 4b), indicative of the combined electrochemical behaviors including Na ion intercalation/de-intercalation at NF@C-650 negative electrode and BF_4_^−^ anion adsorption/desorption at AC positive electrode. Such the battery-capacitor feature demonstrated the successful match between faradaic NF@C-650 and non-faradaic AC. To simultaneously gain the optical charge and kinetics match between positive electrodes and negative electrodes, we implemented the various thickness ratios of NF@C-650 and AC ranging from 1:0.65 to 1:2.6. As a consequence, the resultant NIMSCs delivered gradually increased areal capacitance from 7.01 mF cm^−2^ (1:0.65), 11.2 mF cm^−2^ (1:1.3), 12.1 mF cm^−2^ (1:1.95) to 14.2 mF cm^−2^ (1:2.6) at 20 μA cm^−2^ as increasing the AC thickness (Fig. S19a). Remarkably, the NIMSCs with a thickness ratio of 1:1.3 showed the highest volumetric capacitance of 9.8 F cm^−2^ at 20 μA cm^−2^ (Fig. S19b), compared to NIMSCs (1:0.65) with 8.6 F cm^−3^, NIMSCs (1:1.95) with 8.2 F cm^−3^ and NIMSCs (1:2.6) with 7.9 F cm^−3^. In addition, at high current density of 500 μA cm^−2^, the highest areal capacitance of 5.6 mF cm^−2^ (1:1.3) corresponding to the volumetric capacitance of 4.8 F cm^−3^ was achieved (Fig. 4c), suggestive of robust rate capability with 50% of initial capacitance. Therefore, it is unraveled that the optimal thickness ratio of 1:1.3 for negative electrodes to positive electrode was adopted to fabricate high-performance NIMSCs with favorable capacity and kinetics double-matching.

To understand the excellent rate capability of NIMSCs, the ion reaction kinetics was analyzed by CV measurements. As shown in Fig. 4d, CV curves showed a pair of redox peaks between 2.5 and 3.5 V at 1–10 mV s^−1^, indicative of Na ion insertion/extraction at NF@C-650 electrodes. Further, the surface-controlled pseudocapacitive behavior and diffusion-controlled faradaic process were distinguished by the equation of *i* = *aν*^*b*^ [54], where *ν* denotes the scan rate, *i* represents the peak current, *a* and *b* are variable coefficients [55]. Generally, the diffusion-controlled process was confirmed when the *b* = 0.5, while the *b* = 1 demonstrated the surface-controlled behavior. It is calculated that the *b* values were 0.836 and 0.977 for anodic and cathodic peaks, respectively, suggesting that the charge storage behavior in NF@C-650//AC-NIMSCs is the dominant surface pseudocapacitive contribution (Fig. 4e). Furthermore, the capacitance proportion were quantitatively performed by the following equation: *i* = *k*_*1*_*ν* + *k*_*2*_*v*^*1/2*^ [56], where *k*_*1*_ and *k*_*2*_ are the coefficients. As increasing scan rates from 1 to 10 mV s^−1^, the capacitive contribution of the total capacitance was continuously increased from 63.7 to 83.5% (Figs. 4f and S20). In consequence, it is demonstrated that the high rate capability of NF@C-650//AC-NIMSCs mainly derives from the dominant capacitive-controlled kinetics behaviors.

It should be mentioned that the NF@C-650//AC-NIMSCs can offer outstanding areal capacitance of 41 mF cm^−2^ at 50 μA cm^−2^ through increasing the electrode thickness to 40 μm (Fig. S21), higher than the reported hybrid ion micro-supercapacitors, such as potassium ion micro-supercapacitor (12.6 mF cm^−2^) [57]. Such remarkable results are ascribed to the fast reaction kinetics of elaborate electrode materials and highly conductive channels in microelectrodes. More importantly, the NF@C-650//AC-NIMSCs showed good long-term cycling stability with a decent capacitance retention of 75% after 8,000 continuous cycles at 300 μA cm^−2^ (Fig. 4g). As shown in the Ragone plot (Fig. 4h), it was notable that the NIMSCs delivered high areal energy density of 60.7 μWh cm^−2^, which greatly outnumbered the reported micro-supercapacitors, such as graphene (0.0014 μWh cm^−2^) [43], graphene/PEDOT (0.089 μWh cm^−2^) [44], spray-coating graphene (0.028 μWh cm^−2^) [44], and 3D printing MXene (8.4 μWh cm^−2^) [45]. Morevoer, the NIMSCs exhibited a maximum power density of 1512 μW cm^−2^ at 14.3 μWh cm^−2^.

### Integration of NIMSCs

**Fig. 5:**
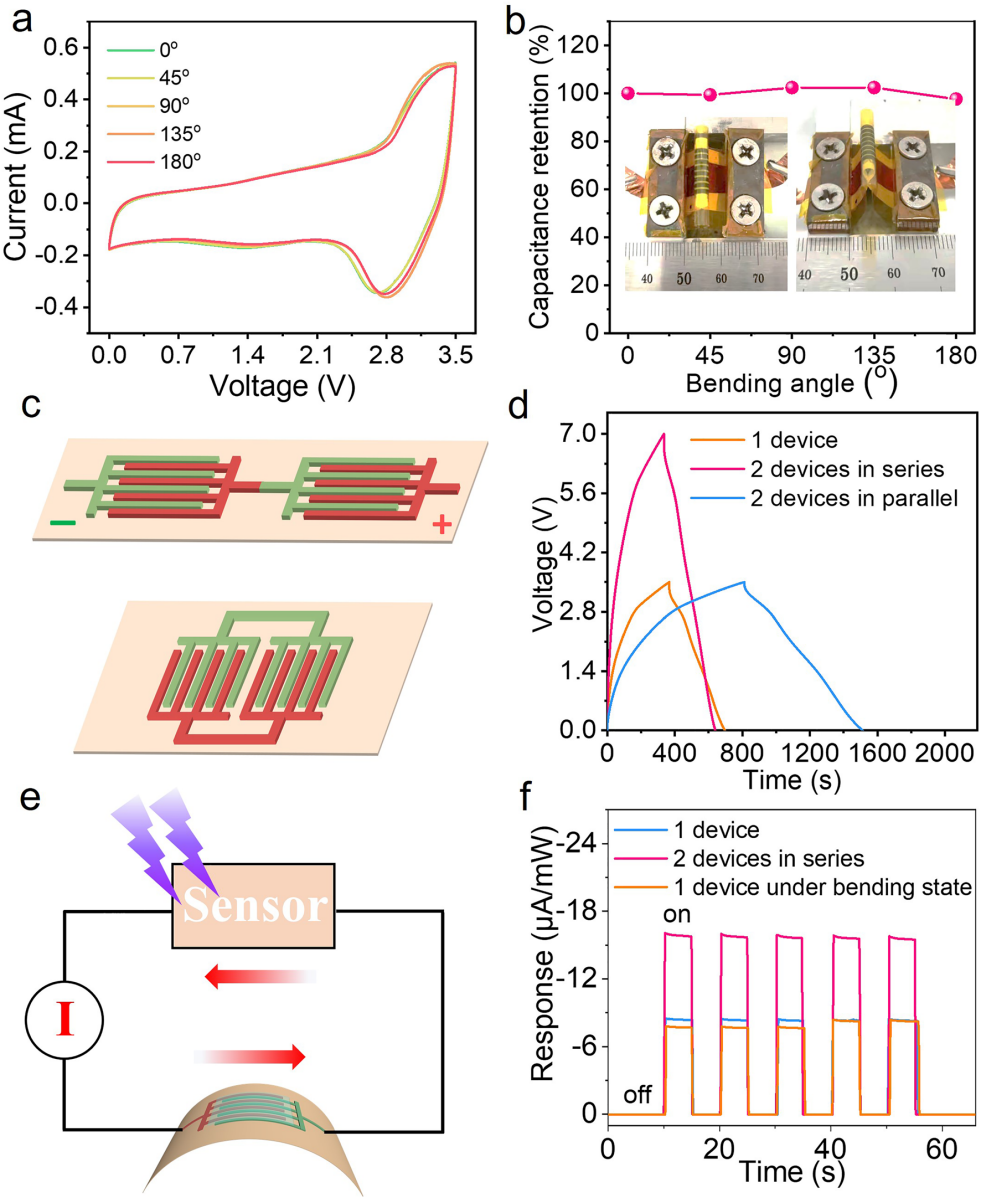
Flexibility and integration performance of NF@C-650//AC-NIMSCs. **a** CV curves of NIMSCs obtained at different bending states at 20 mV s^−1^. **b** Capacitance retention of NIMSCs under various bending angles. Insets are the photographs of the NIMSCs at bending states. **c** Schematic of the serially-connected (top) and parallely-connected (bottom) NIMSCs. **d** GCD profiles at 75 μA cm^−2^ of integrated NIMSCs connected in parallel and in series from 1 to 2 cells. **e** The equivalent circuit of the ultraviolet sensor integrated system powered by flexible NIMSCs. **f** Normalized response current of the ultraviolet sensor

To further cater for flexible microelectronics, we evaluated the electrochemical performance of NF@C-650//AC-NIMSCs under various bending angels from 0 to 180° (Fig. 5a). Remarkably, the NIMSCs tested at the scan rate of 20 mV s^−1^ showed slight changed CV curves and hold a stable capacitance output without capacitance deterioration from the flat state to a high bending angle of 180° (Fig. 5b), indicative of outstanding structual integrity and mechanical flexibility of NF@C-650//AC-NIMSCs. To accommodate different requirements of microelectronics for the voltage and current, we constructed two parallelly or serially connected NIMSCs to boost the capacitance or voltage output without the requirement of metal-based interconnections (Fig. 5c). As shown in Fig. 5d, the capacitance related to current and discharge time of the integrated NIMSCs proportionally increased while the voltage kept unchanged by consecutively connecting cells in parallel. Besides, the operating voltage exhibited a linear increase from 3.5 V for one cell to 7 V for two serially-connected cells. As examplified, the single and two serially-connected NIMSCs can easily power an ultraviolet sensor (Fig. 5e, f), in which the response current was proportionally increased by increasing the serially-connected cell. Notably, there was no obvious change in the current when the NIMSC was in the bending state, witnessing its functional role in flexible power source. These results indicated that the NIMSCs hold great prospect for application for flexible microelectronics devices to meet customized flexible microelectronics.

## Conclusions

In summary, we have developed novel hierarchical structured Nb_2_O_5_ microflowers with porous and ultrathin nanosheets and thin carbon-layer, boosting Na ion storage. The elaborate NF@C-650 delivered superior capacity and rate capability. The outstanding electrochemical performance of NF@C-650 was ascribed to the following advantages: (i) ultrathin nanosheets shortened ion/electron diffusion pathway; (ii) abundant pore structures boosted electrolyte infiltration and Na ion transport; (iii) unique carbon layer on the surface of Nb_2_O_5_ improved the electrical conductivity and interface stability. By matching the NF@C-650 negative electrode and AC positive electrode, the as-obtained planar NIMSCs showed superior areal capacitance of 41 mF cm^−2^, as well as excellent areal energy density of 60.7 μWh cm^−2^, outstanding cycling stability and flexibility. Therefore, this work demonstrates that well-designed electrode structure with fast reaction kinetics becomes an appropriate approach for planar hybrids ion micro-supercapacitors.

## Supplementary Information

Below is the link to the electronic supplementary material.

Supplementary file1 (DOCX 4175 KB)
