# Supplementary material for: Hierarchically Structured Nb2O5 Microflowers with Enhanced Capacity and Fast-Charging Capability for Flexible Planar Sodium Ion Micro-Supercapacitors
Source: Nanomicro Lett. 2024 Jan 4;16:67. doi: 10.1007/s40820-023-01281-5 (PMC10766898; doi:10.1007/s40820-023-01281-5)
Supplement: Supplementary file 1 — Supplementary file1 (DOCX 4175 KB) [file 40820_2023_1281_MOESM1_ESM.docx]

Supporting Information for

**Hierarchically Structured Nb_2_O_5_ Microflowers with Enhanced Capacity and Fast-Charging Capability for Flexible Planar Sodium Ion Micro-Supercapacitors**

Jiaxin Ma,^1,2^ Jieqiong Qin,^3^ Shuanghao Zheng,^1,4,^* Yinghua Fu,^1,5^ Liping Chi,^1^ Yaguang Li,^6^ Cong Dong,^1,5^ Bin Li,^1,5^ Feifei Xing,^1,5^ Haodong Shi,^1,4^ and Zhong-Shuai Wu^1,4,^*

^1^ State Key Laboratory of Catalysis, Dalian Institute of Chemical Physics, Chinese Academy of Sciences, 457 Zhongshan Road, Dalian 116023, China

^2^ School of Materials Science and Engineering, Zhengzhou University, Zhengzhou 450001, China

^3^ College of Science, Henan Agricultural University, No. 63 Agricultural Road, Zhengzhou 450002, China

^4^ Dalian National Laboratory for Clean Energy, Chinese Academy of Sciences, 457 Zhongshan Road, Dalian 116023, China

^5^ University of Chinese Academy of Sciences, 19A Yuquan Road, Shijingshan District, Beijing 100049, China

^6^ Hebei Key Lab of Optic-electronic Information and Materials, The College of Physics Science and Technology, Institute of Life Science and Green Development, Hebei University, Baoding 071002, China

*Corresponding author. E-mail: shuanghao_zheng@dicp.ac.cn; wuzs@dicp.ac.cn

**Materials Characterization**

The morphology was confirmed through scanning electron microscopy (SEM, JSM-7800F), transmission electron microscopy (TEM, JEM-F200). The structure of the active materials was analyzed by X-ray diffraction (XRD) (SmartLab, 5~90°), Raman spectroscopy (LabRAM HR 800 Raman spectrometer, 632 nm), physical adsorption instrument (Micromeritics APSP 2460) and X-ray photoelectron spectroscopy (XPS, Thermofisher Escalab 250 Xi^+^), electron paramagnetic resonance (EPR, Bruker A200) and thermogravimetric (TG, Diamond).

**Electrochemical Measurement**

The half-cell Nb_2_O_5_ was assembled in coin cells (CR2025) with sodium foil as the counter and reference electrodes at an argon filled glove box. The working electrodes (12 mm in diameter) consist of Nb_2_O_5_, carbon black, and polyvinylidene fluoride with a weight ratio of 7:2:1. The average mass loading of active materials is about 1-1.2 mg/cm^2^. The electrochemical performances were evaluated in 1.0 M NaClO_4_ electrolyte in ethylene carbonate and dimethyl carbonate (1:1 v/v) with 5% fluoroethylene carbonate electrolyte at a potential range between 0.01 and 3.0 V (*vs.* Na/Na^+^). GCD profiles were investigated at a LAND CT3001A battery tester. CV curves at 0.1 mV/s and EIS at a wide frequency range from 0.01 Hz to 100 kHz with an AC amplitude of 5 mV were performed on a CHI 760E electrochemical workstation. For NIMSCs, GCD profiles at various current densities from 20 to 500 μA/cm^2^ were tested by the LAND CT3001A battery tester. CV curves from 1 to 10 mV/s were measured on a CHI 760E electrochemical workstation.

**Calculation**

The areal capacitance and volumetric capacitance of NIMSCs are calculated from GCD profiles according to the following equations (1) and (2) [1]:

$C_{areal}=\frac{It}{A}$ (1)

$C_{volume}=\frac{It}{V}$ (2)

Where $C_{areal}$ (F/m^2^) and $C_{volume}$ (F/cm^3^) are the areal capacitance and volumetric capacitance, respectively, *I* (A) is the discharge current, *t* (s) is the discharge time, *A* (cm^2^) and *V* (cm^3^) are the area and volume of the whole microelectrodes, respectively.

The areal energy density and power density of NIMSCs are evaluated by the formula (3) and (4):

$E_{areal}=\frac{I\int Udt}{A}$ (3)

$P_{areal}=\frac{E_{areal}}{t}\times3600$ (4)

Where *U* (V) is voltage,$E_{areal}$ (mWh/cm^2^) and $P_{areal}$ (mW/cm^2^) are areal energy and power density, respectively.


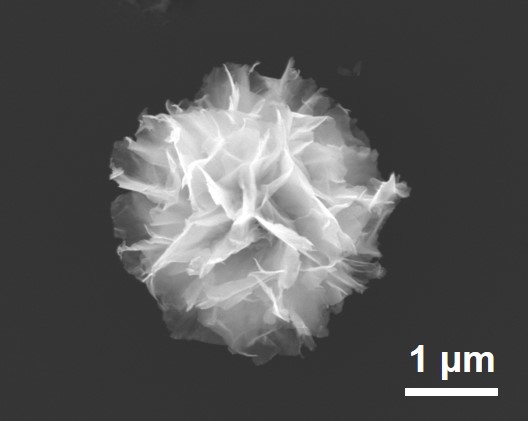


**Fig. S1**. SEM image of NF-500.

**Fig S2**. XRD patterns (**a**) and Raman spectra (**b**) of flower-like precursor and Nb_2_O_5_ microflowers at different annealed temperature.


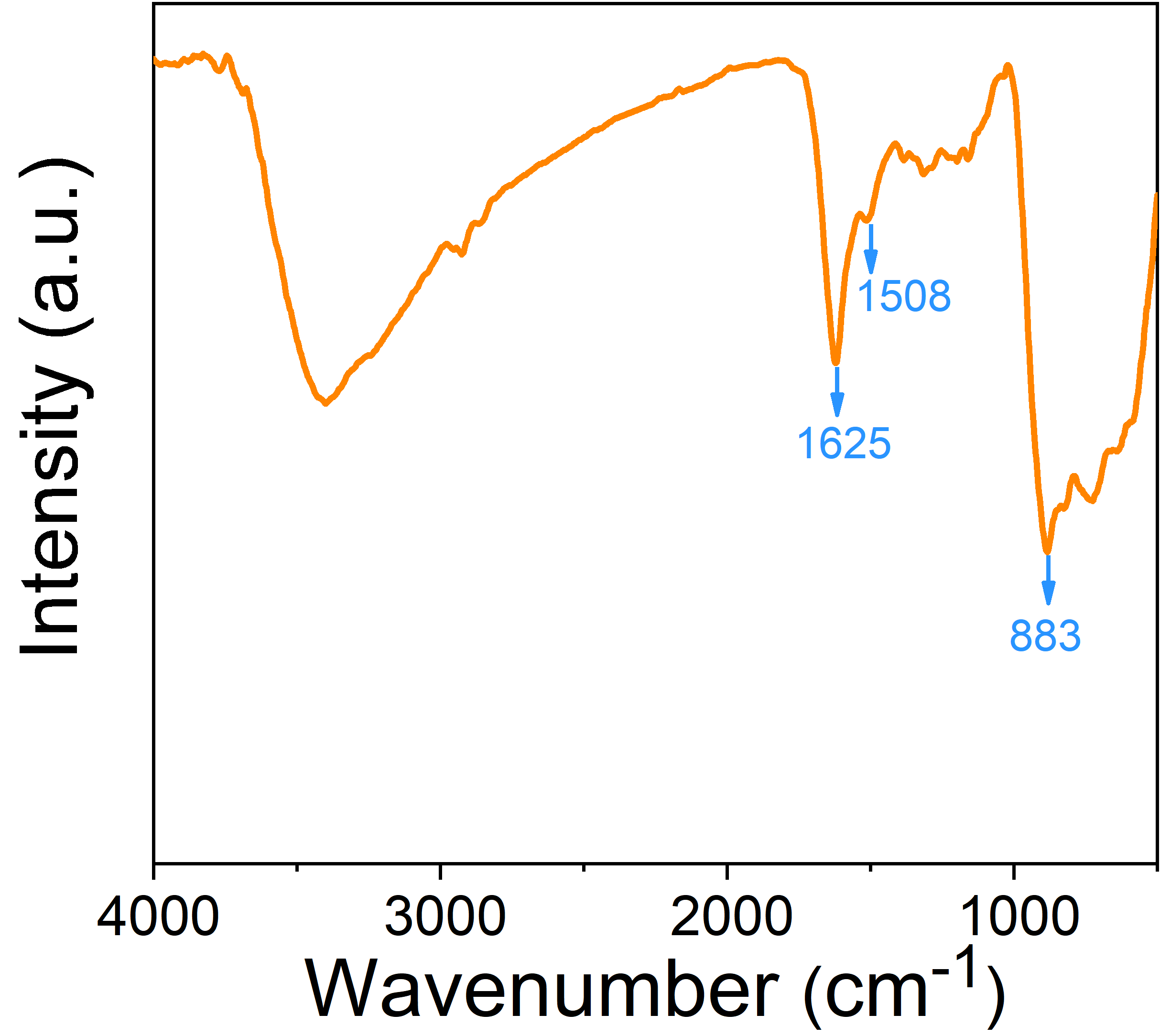


**Fig. S3**. FTIR spectrum of NF-650 microflowers coated by polydopamine.


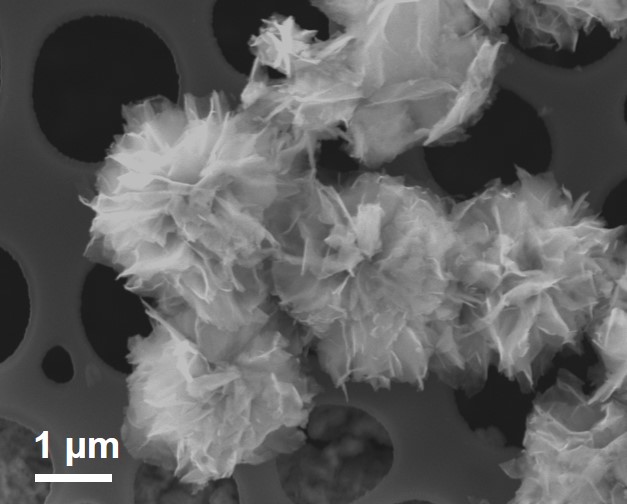


**Fig. S4**. SEM image of flower-like precursor coated with polydopamine.


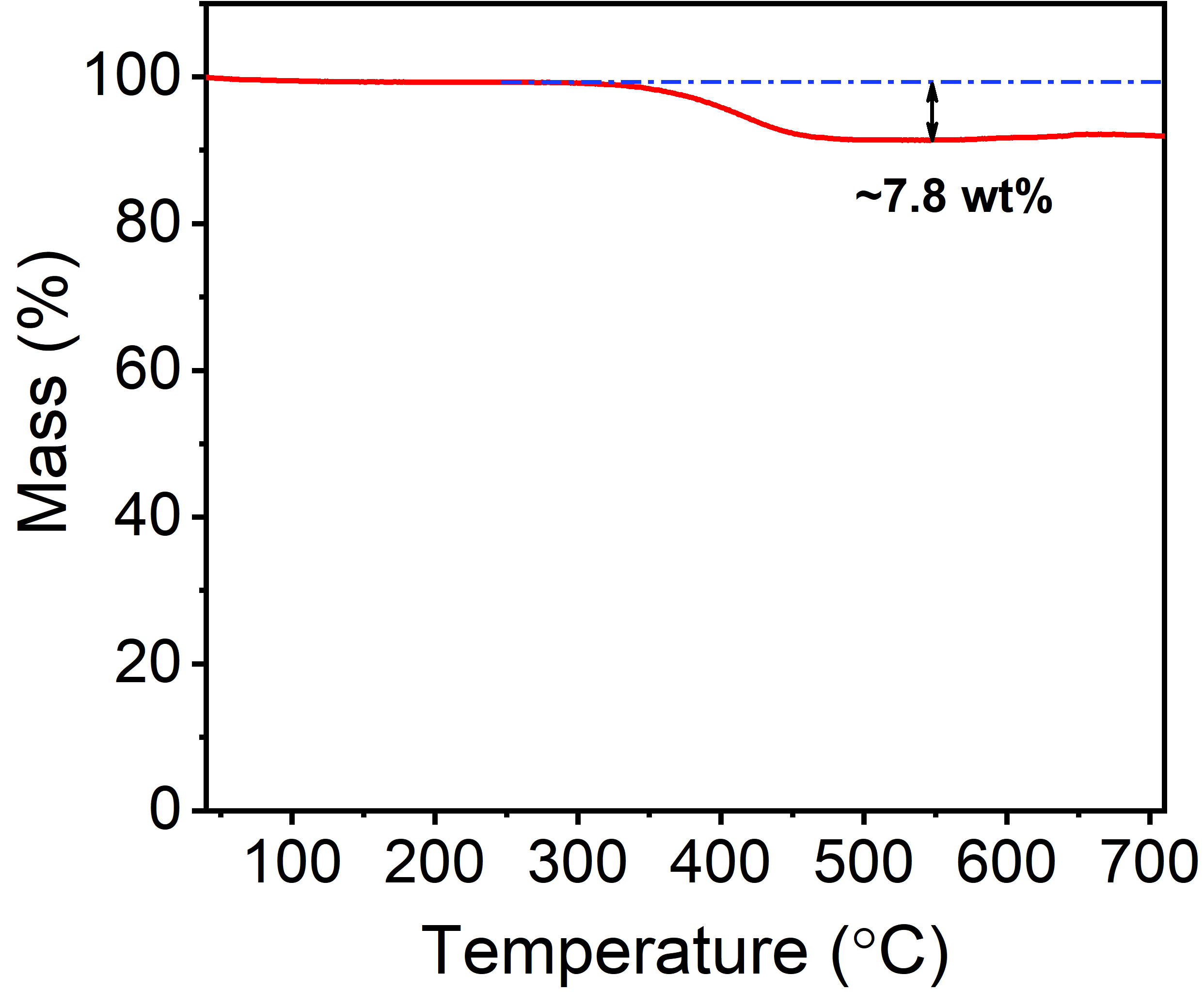


**Fig. S5**. TG curve of NF@C-650.


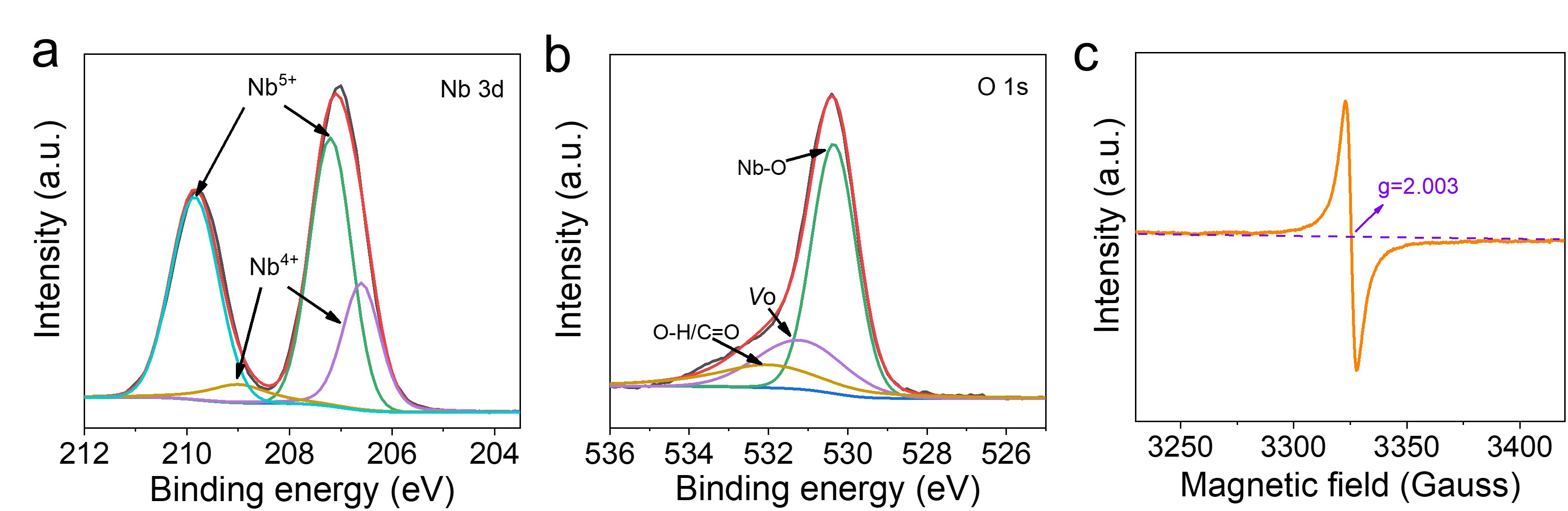


**Fig. S6**. **a,b** Nb 3d (**a**) and O 1s (**b**) core-level XPS spectra of NF@C-650. **c** EPR spectrum of NF@C-650.

**
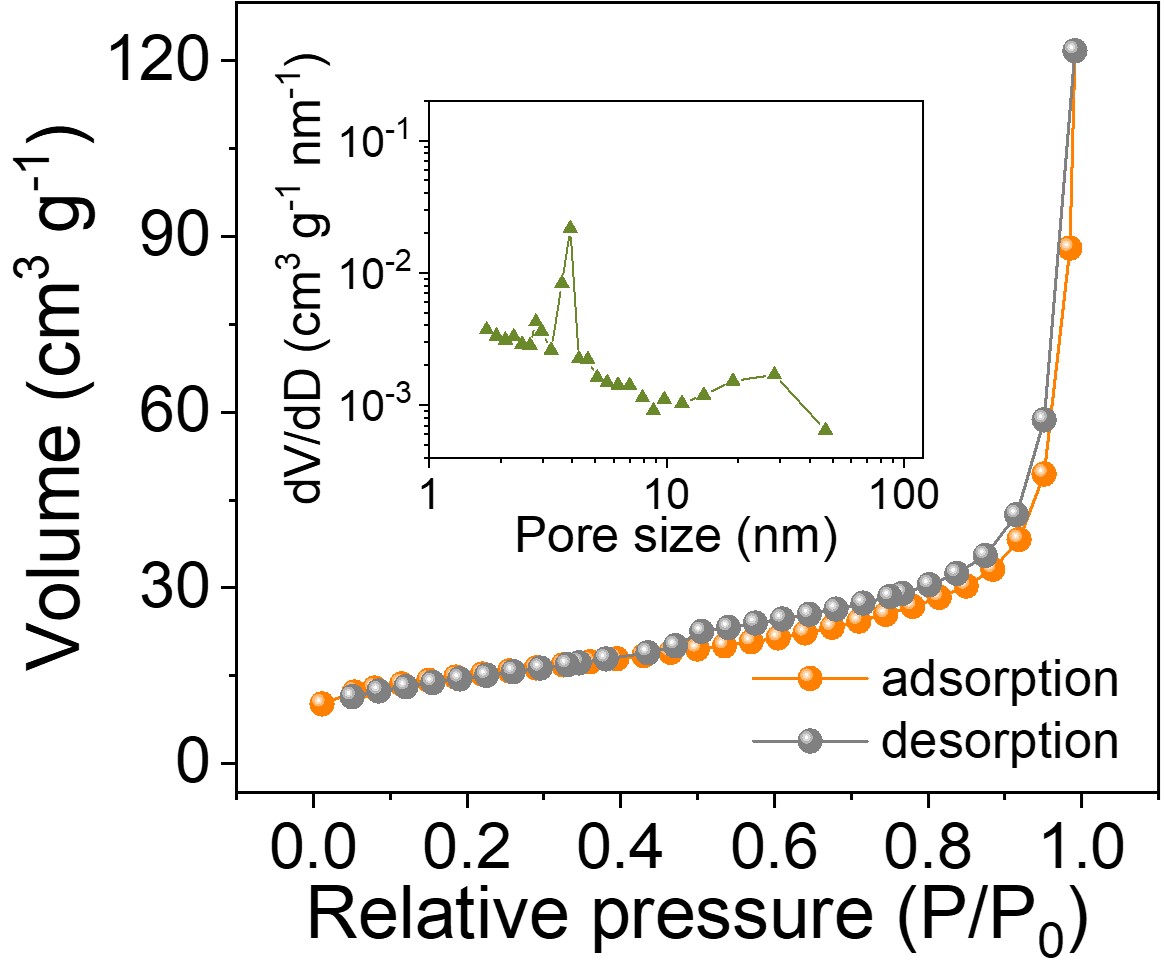
**

**Fig. S7**. N_2_ adsorption/desorption isotherm and corresponding pore size distribution (Inset) of NF@C-650.


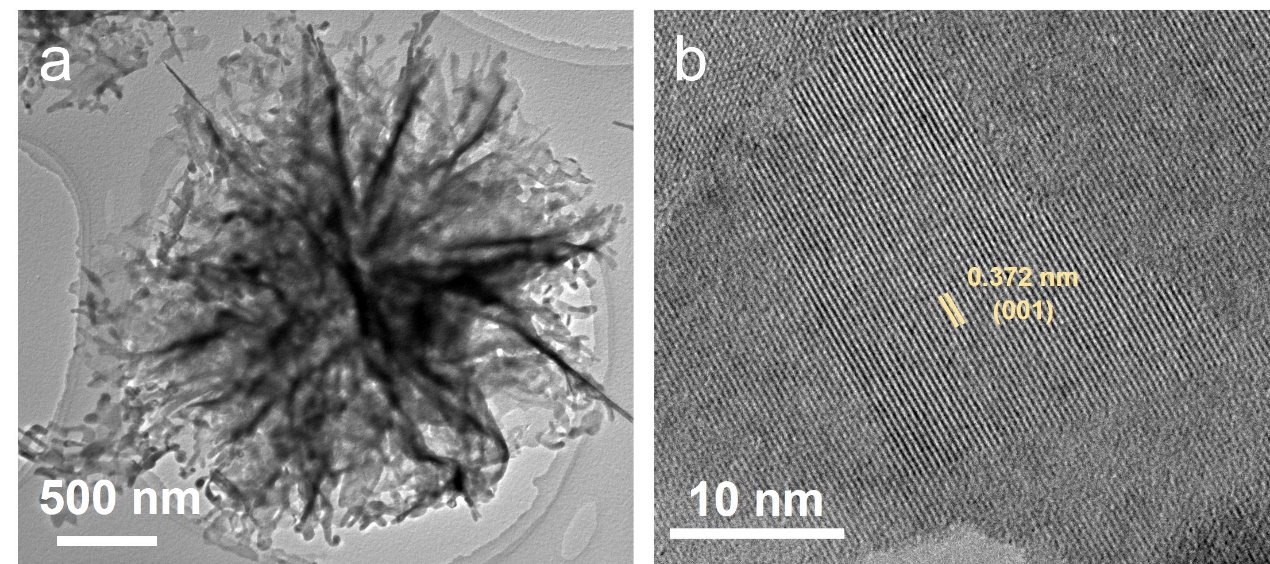


**Fig. S8**. **a,b** TEM image (**a**) and HRTEM image (**b**) of NF-650.


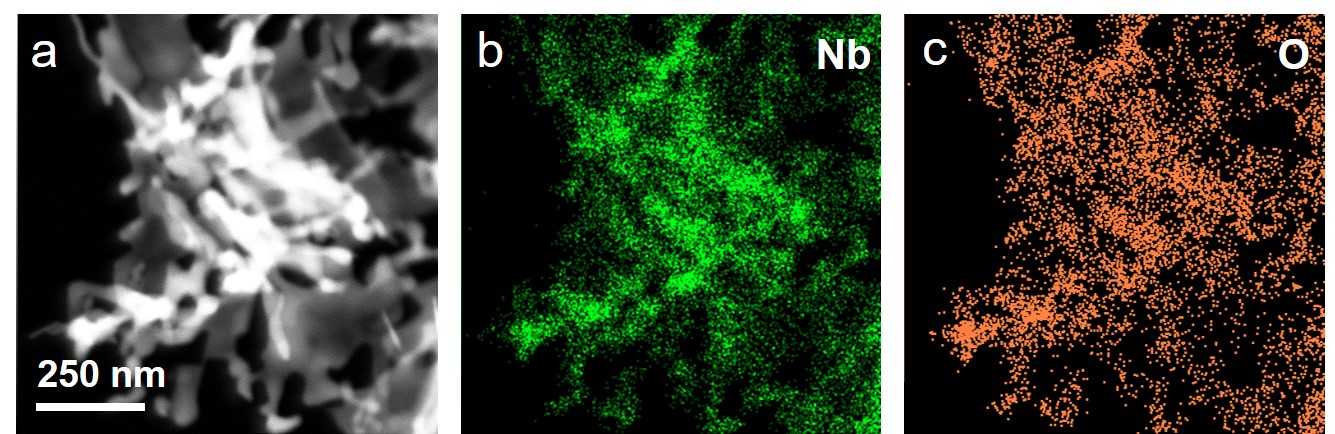


**Fig. S9**. **a-c** STEM image (**a**) and corresponding EDS mappings (**b,c**) of NF-650.


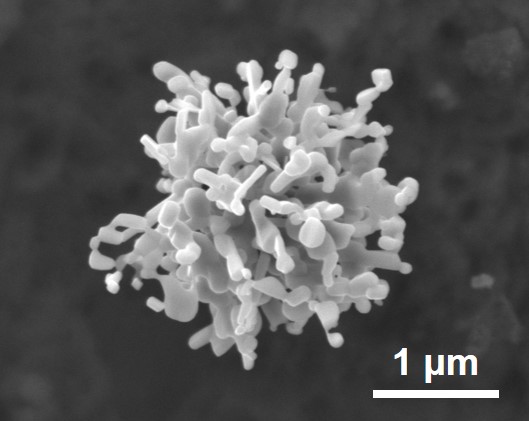


**Fig. S10**. SEM image of NF-800.


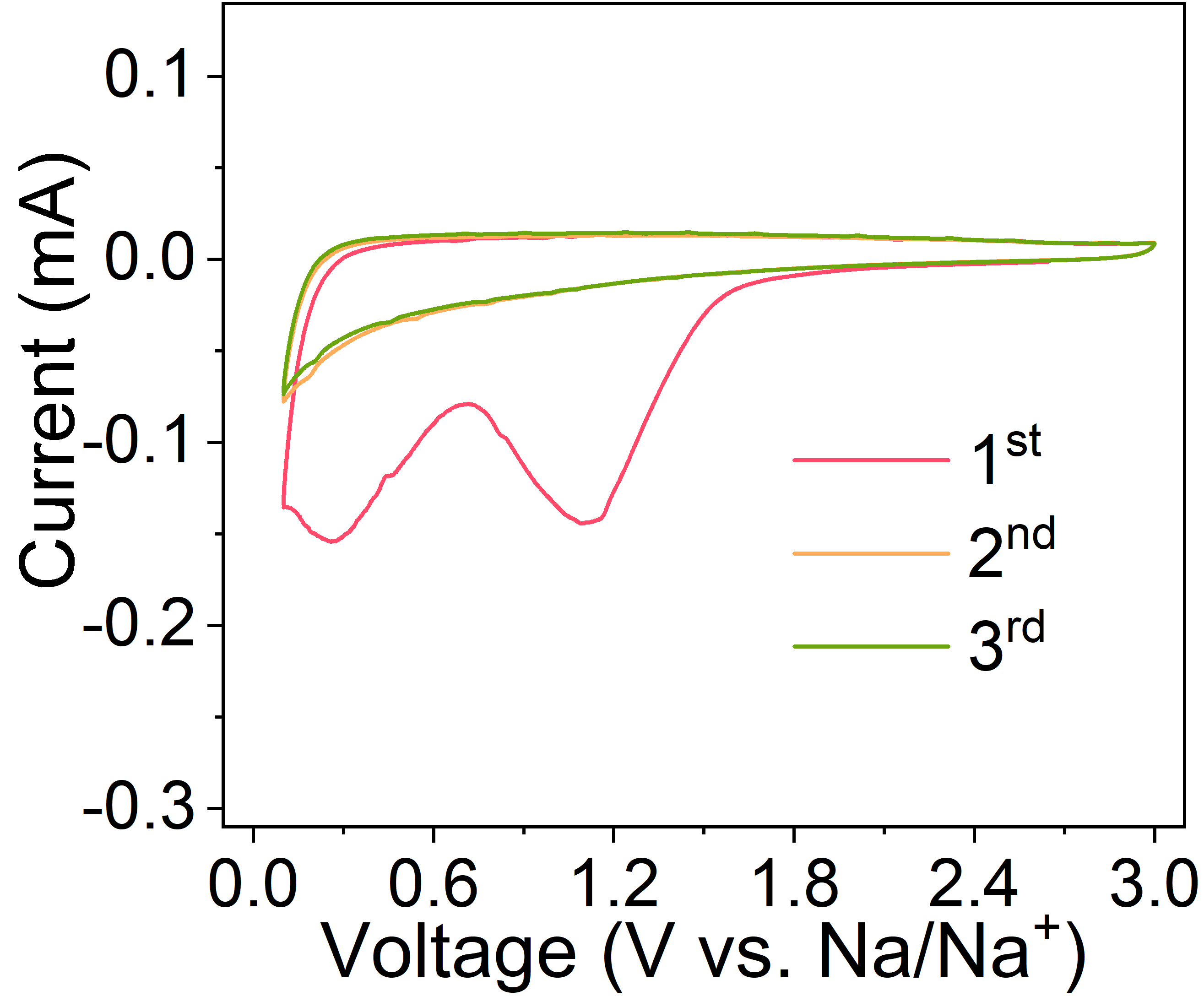


**Fig. S11**. CV curves of NF@C-650 tested at 0.1 mV/s at a potential window of 0.01-3.0 V (*vs.* Na/Na^+^).

**Fig. S12**. **a,b** GCD profiles of Nb_2_O_5_ microflowers tested at 0.25 C (**a**) and 20 C (**b**).


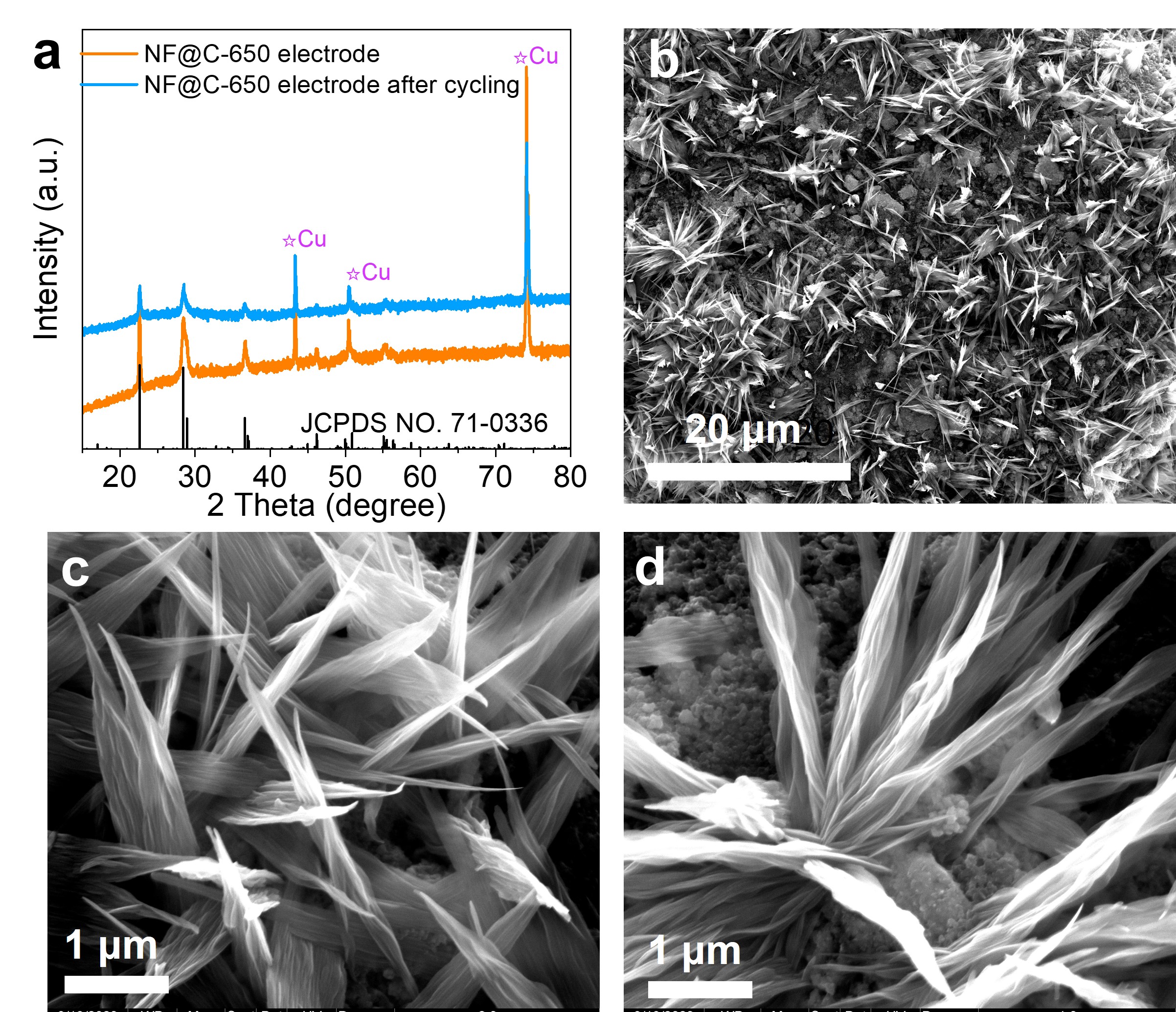


**Fig. S13**. **a** XRD patterns of NF@C-650 electrodes before and after 1000 cycles tested at 20 C. **b-d** SEM images of NF@C-650 electrodes tested at 20 C after 1000 cycles.


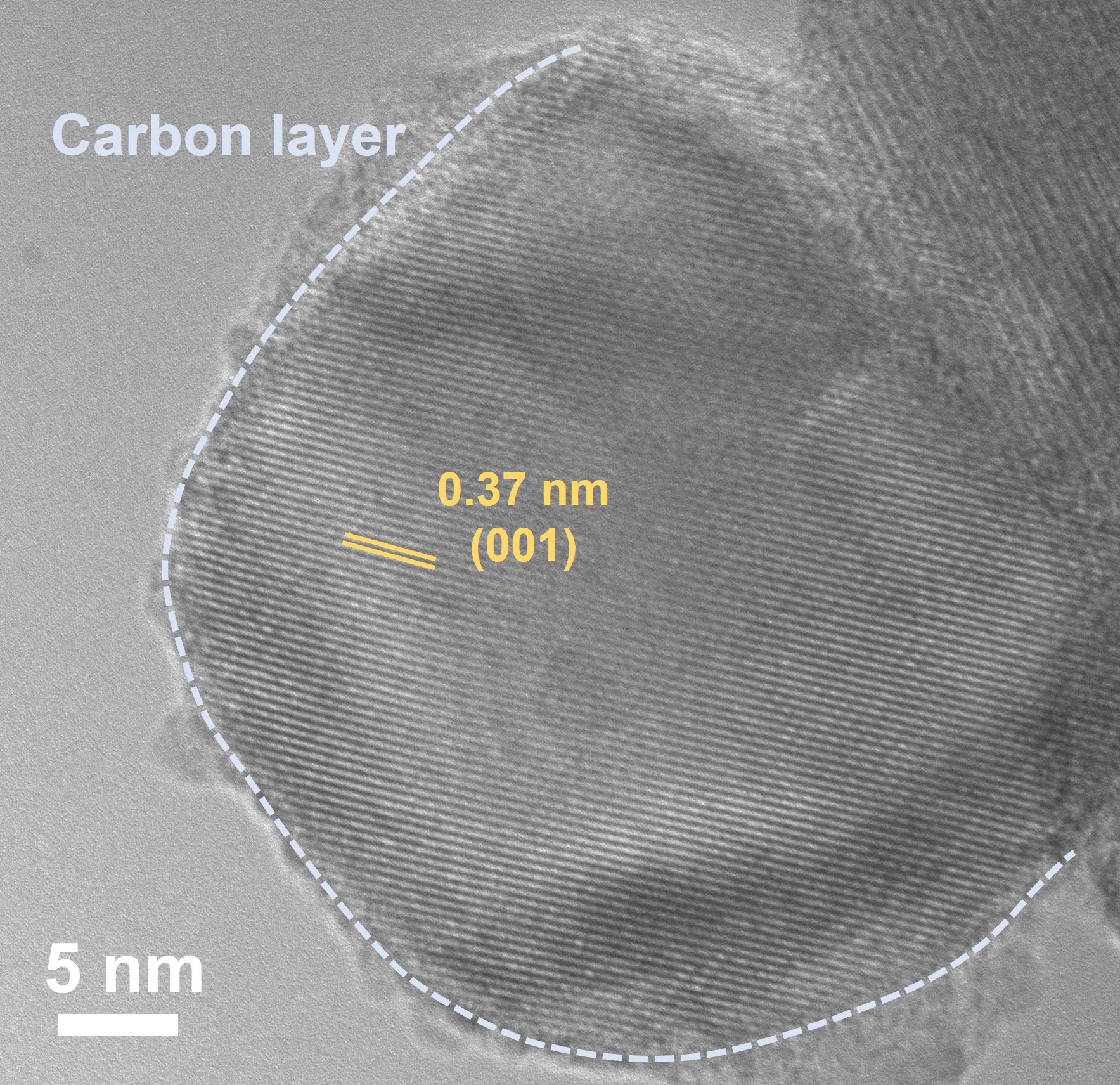


**Fig. S14**. HRTEM image of NF@C-650 tested at 20 C after 1000 cycles.


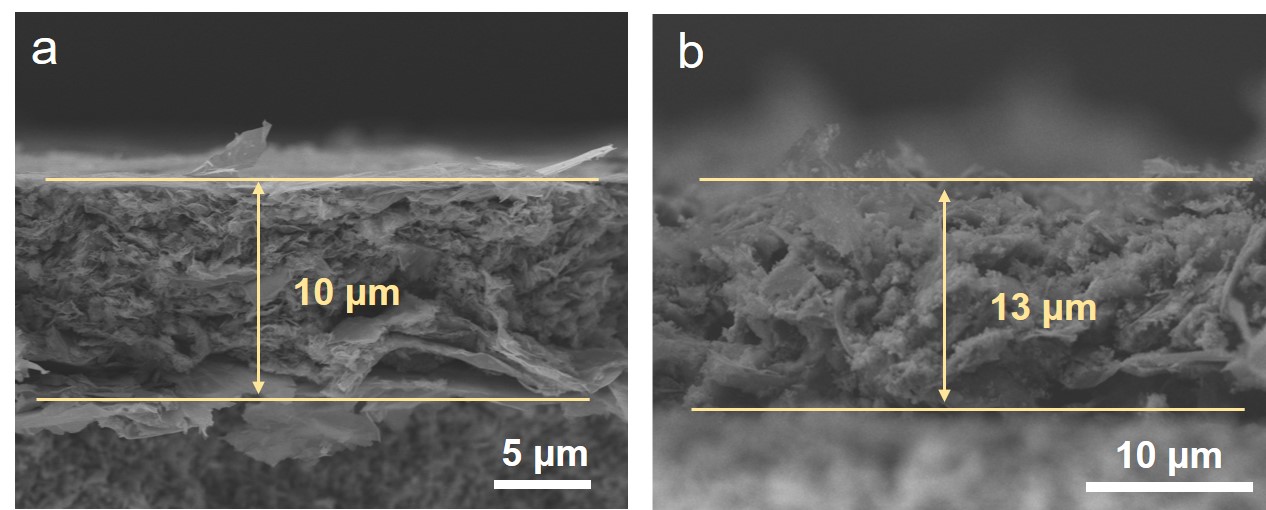


**Fig. S15**. **a,b** Cross-section SEM images of Nb_2_O_5_ negative electrode (**a**) and AC positive electrode (**b**).


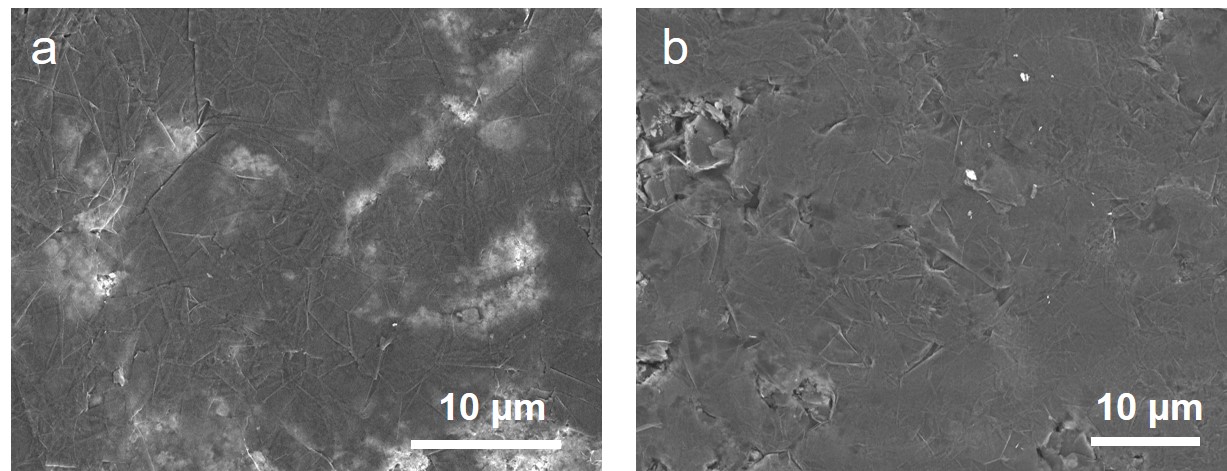


**Fig. S16**. **a,b** SEM images of Nb_2_O_5_ negative electrode (**a**) and AC positive electrode (**b**).

**Fig. S17**. Electrical conductivity of Nb_2_O_5_ negative electrode (**a**) and AC positive electrode (**b**).


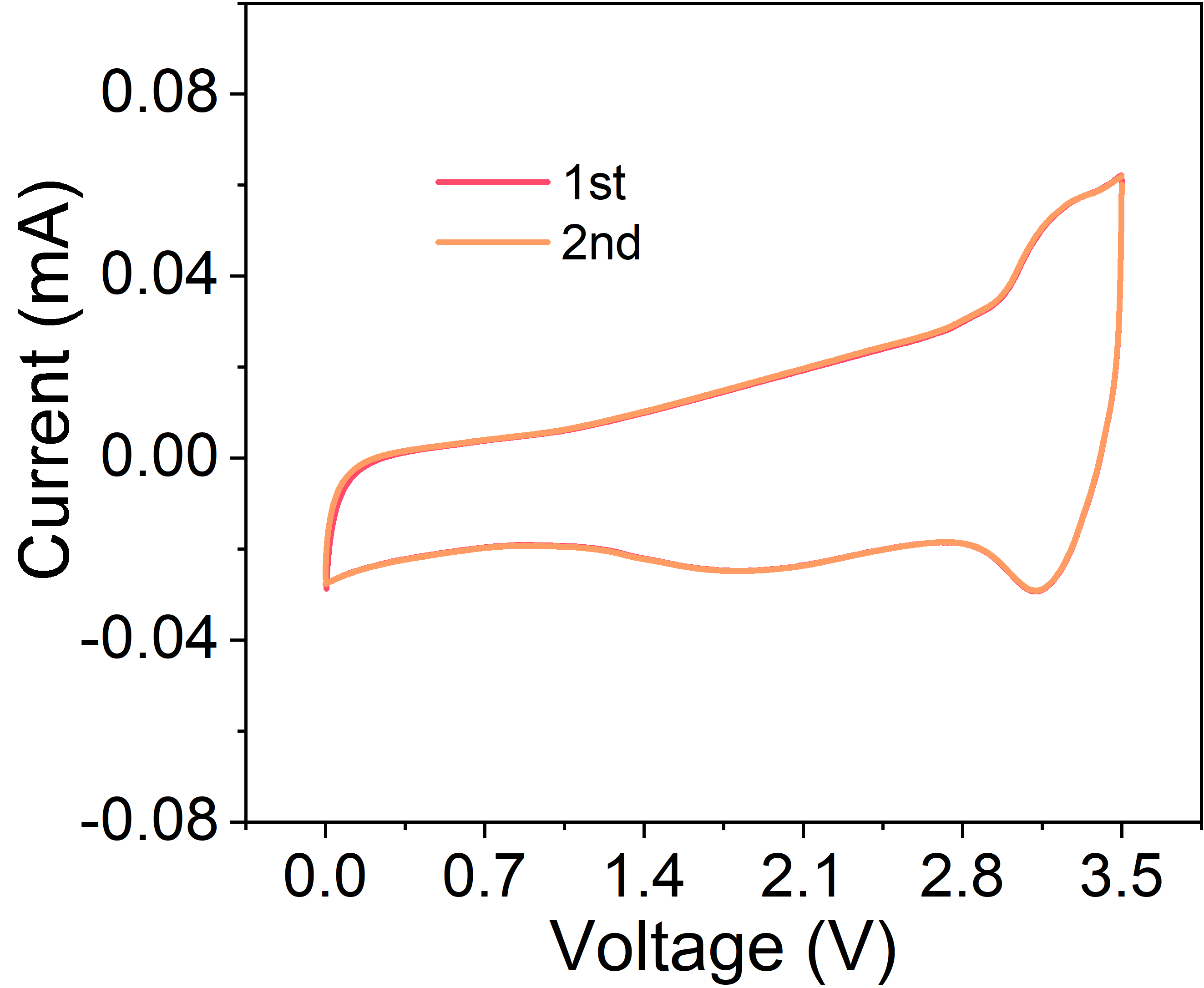


**Fig. S18**. CV curves of NIMSCs tested 2 mV/s.

**Fig. S19**. Rate capability of NIMSCs under different thickness ratios of NF@C-650 : AC. **a** Areal capacitance and **b** volumetric capacitance at different current densities.


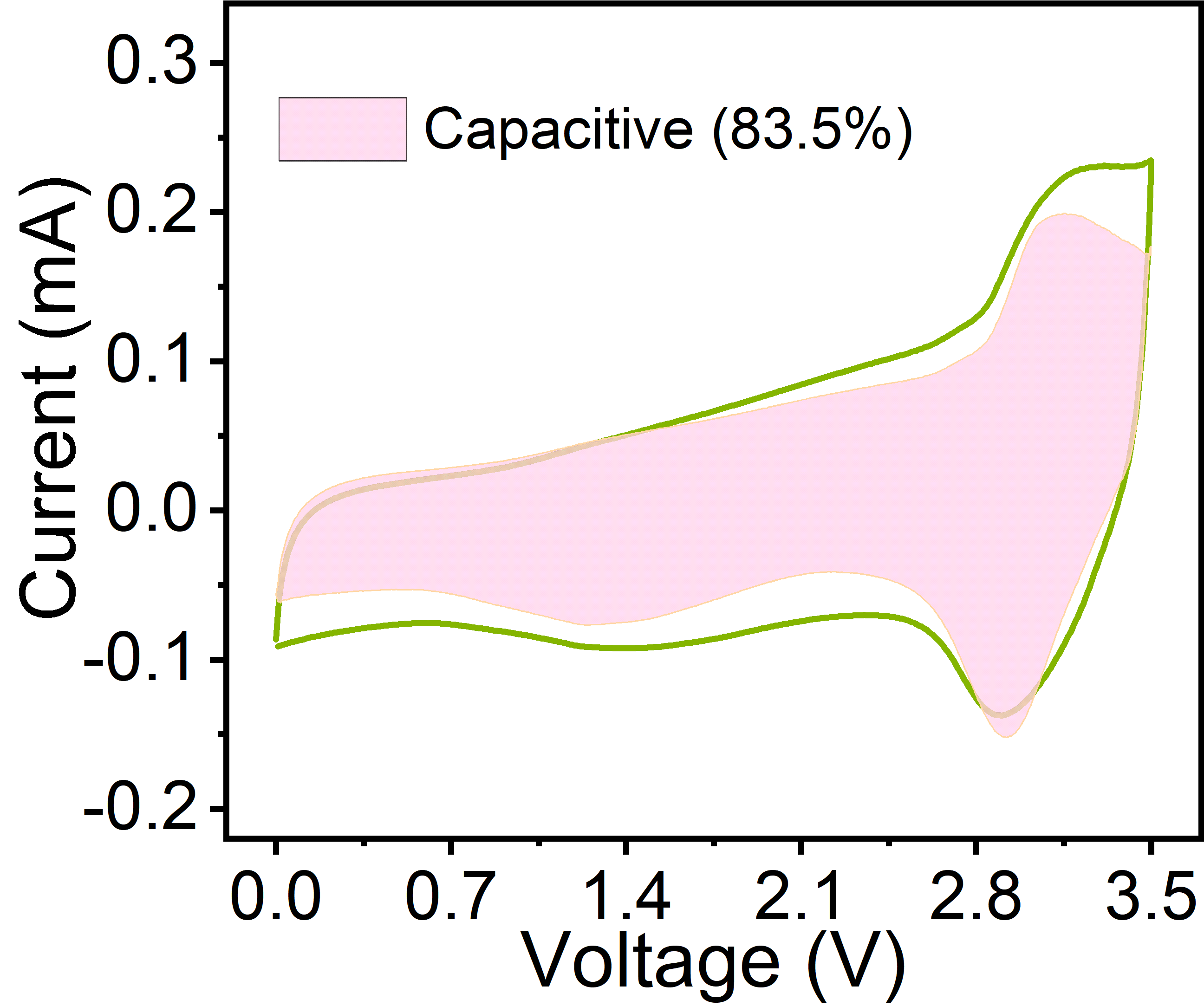


**Fig. S20**. Capacitive and diffusion-controlled capacitance of NF@C-650//AC-NIMSCs at 10 mV/s.

**Fig. S21**. **a** GCD profiles of NIMSCs measured at 50-150 μA/cm^2^. **b** Areal capacitance of NIMSCs calculated from GCD profiles at 50-500 μA/cm^2^.

**Table S1**. The comparison of the properties of our NIMSCs and reported MSCs.

| Devices | Configuration | Methods | Voltage  (V) | Areal capacitance (mF/cm^2^) | Energy density  (μWh/cm^2^) | Ref. |
| --- | --- | --- | --- | --- | --- | --- |
| Nb_2_O_5_//AC NIMSCs | Interdigital | Mask-assisted filtration | 3.5 V | 41 | 60.7 | This work |
| GO/CNT//GO/CNT MSCs | Interdigital | Drop-casting | 0.8 V | 68.6 | 6.1 | [2] |
| Carbon//carbon MSCs | Interdigital | Inkjet printing | 1.0 V | 3.9 | 7.7 | [3] |
| GO//GO MSCs | Interdigital | 3D printing | 1.0 V | 128.4 | 17.8 | [4] |
| CNT@Mn/V// CNT@Mn/V MSCs | Interdigital | photolithography | 0.8 V | 11.8 | 6.58 | [5] |

AC: activated oxide, GO: graphene oxide, CNT: carbon nanotube.

**References**

1. Zheng, S.; Wang, S.; Dong, Y.; Zhou, F.; Qin, J., et al., All-solid-state planar sodium-ion microcapacitors with multidirectional fast ion diffusion pathways. Adv. Sci. **6** (23), 1902147 (2019). <https://doi.org/10.1002/advs.201902147>

2. Yuan, M.; Luo, F.; Wang, Z.; Yu, J.; Li, H., et al., Smart wearable band-aid integrated with high-performance micro-supercapacitor, humidity and pressure sensor for multifunctional monitoring. Chem. Eng. J. **453**, (2023). <https://doi.org/10.1016/j.cej.2022.139898>

3. Bräuniger, Y.; Lochmann, S.; Grothe, J.; Hantusch, M.; Kaskel, S., Piezoelectric inkjet printing of nanoporous carbons for micro-supercapacitor devices. ACS Appl. Energy Mater. **4** (2), 1560-1567 (2021). <https://doi.org/10.1021/acsaem.0c02745>

4. Chen, Y.; Guo, M.; Xu, L.; Cai, Y.; Tian, X., et al., In-situ selective surface engineering of graphene micro-supercapacitor chips. Nano Research **15** (2), 1492-1499 (2021). <https://doi.org/10.1007/s12274-021-3693-4>

5. Park, H.; Song, C.; Jin, S. W.; Lee, H.; Keum, K., et al., High performance flexible micro-supercapacitor for powering a vertically integrated skin-attachable strain sensor on a bio-inspired adhesive. Nano Energy **83**, (2021). <https://doi.org/10.1016/j.nanoen.2021.105837>
